# Interleukin-6 Deficiency Attenuates Retinal Ganglion Cell Axonopathy and Glaucoma-Related Vision Loss

**DOI:** 10.3389/fnins.2017.00318

**Published:** 2017-05-31

**Authors:** Franklin D. Echevarria, Cathryn R. Formichella, Rebecca M. Sappington

**Affiliations:** ^1^Neuroscience Graduate Program, Vanderbilt UniversityNashville, TN, United States; ^2^Department of Ophthalmology and Visual Sciences, Vanderbilt University School of MedicineNashville, TN, United States; ^3^Vanderbilt Eye Institute, Vanderbilt University Medical CenterNashville, TN, United States; ^4^Department of Pharmacology, Vanderbilt University School of MedicineNashville, TN, United States

**Keywords:** interleukin-6, cytokine, retinal ganglion cell, glaucoma, axonopathy, microbead, cornea, wound healing

## Abstract

The pleotropic cytokine interleukin-6 (IL-6) is implicated in retinal ganglion cell (RGC) survival and degeneration, including that associated with glaucoma. IL-6 protects RGCs from pressure-induced apoptosis *in vitro*. However, it is unknown how IL-6 impacts glaucomatous degeneration *in vivo*. To study how IL-6 influences glaucomatous RGC axonopathy, accompanying glial reactivity, and resultant deficits in visual function, we performed neural tracing, histological, and neurobehavioral assessments in wildtype (B6;129SF2/J; WT) and IL-6 knock-out mice (B6;129S2-*IL6*^*t*^^m1kopf^/J; *IL-6*-/-) after 8 weeks of unilateral or bilateral microbead-induced glaucoma (microbead occlusion model). IOP increased by 20% following microbead injection in both genotypes (*p* < 0.05). However, deficits in wound healing at the site of corneal injection were noted. In WT mice, elevated IOP produced degenerating axon profiles and decreased axon density in the optic nerve by 15% (*p* < 0.01). In *IL-6*-/- mice, axon density in the optic nerve did not differ between microbead- and saline-injected mice (*p* > 0.05) and degenerating axon profiles were minimal. Preservation of RGC axons was reflected in visual function, where visual acuity decreased significantly in a time-dependent manner with microbead-induced IOP elevation in WT (*p* < 0.001), but not *IL-6*-/- mice (*p* > 0.05). Despite this preservation of RGC axons and visual acuity, both microbead-injected WT and *IL-6*-/- mice exhibited a 50% decrease in anterograde CTB transport to the superior colliculus, as compared to saline-injected controls (*p* < 0.01). Assessment of glial reactivity revealed no genotype- or IOP-dependent changes in retinal astrocytes. IOP elevation decreased microglia density and percent retinal area covered in WT mice (*p* < 0.05), while *IL-6*-/- mice exhibited only a decrease in density (*p* < 0.05). Together, our findings indicate that two defining features of RGC axonopathy—axon transport deficits and structural degeneration of axons—likely occur via independent mechanisms. Our data suggest that IL-6 is part of a mechanism that specifically leads to structural degeneration of axons. Furthermore, its absence is sufficient to prevent both structural degeneration of the optic nerve and vision loss. Overall, our work supports the proposition that functional deficits in axon transport represent a therapeutic window for RGC axonopathy and identify IL-6 signaling as a strong target for such a therapeutic.

## Introduction

The pleotropic cytokine interleukin-6 (IL-6) is involved in a variety of central nervous system (CNS) pathologies including injury, infection, and neurodegeneration (Erta et al., 2012). Its classification as either protective or destructive within the CNS continues to be highly contested. Pre-treatment with IL-6 prevents apoptosis in neural cells exposed to a number of physiological stressors *in vitro*, supporting the idea that IL-6 is neuro-protective (Yamada and Hatanaka, 1994; Sappington et al., 2006; Spittau et al., 2012; Fang et al., 2013; Chucair-Elliott et al., 2014). In animal models of CNS disease, loss of IL-6 leads to an overall reduction in the neuroinflammatory response, including reduced expression of other inflammatory cytokines and diminished glial reactivity (Penkowa et al., 1999, 2000, 2001; Clark et al., 2000; Cardenas and Bolin, 2003). Interestingly, the effect on neuronal health is variable, as studies suggest that IL-6 signaling promotes both viability (Yamada and Hatanaka, 1994; Loddick et al., 1998; Zhong et al., 1999; Clark et al., 2000; Cardenas and Bolin, 2003; Inomata et al., 2003; Penkowa et al., 2003; Sappington et al., 2006; Spittau et al., 2012; Fang et al., 2013; Leibinger et al., 2013; Chucair-Elliott et al., 2014) and dysfunction (Campbell et al., 1993; Bluthe et al., 2000; Sparkman et al., 2006; Mukaino et al., 2010; Burton et al., 2011, 2013; Burton and Johnson, 2012) depending on the model of CNS injury. IL-6 mRNA and protein are upregulated near retinal ganglion cells (RGCs) and their axons in rodent models of glaucoma (Sappington and Calkins, 2008; Chidlow et al., 2012; Sims et al., 2012; Wilson et al., 2015). Glaucoma is a neurodegenerative disease characterized by RGC axonopathy and associated with both advanced age and elevated intraocular pressure (IOP) (Calkins, 2012). Like elsewhere in the CNS, the role of IL-6 in RGC axonopathy is unclear. Application of recombinant IL-6 to RGCs *in vitro* prevents pressure-induced apoptosis (Sappington et al., 2006). Similarly, IL-6 appears to protect RGCs and enhance axon regeneration following optic nerve crush (Leibinger et al., 2013, 2016). In contrast, other studies indicate that IL-6 deficiency protects RGCs in models of glutamate excitotoxicity and optic nerve crush (Fisher et al., 2001).

To better elucidate the impact of IL-6 signaling on RGC axonopathy in glaucoma, we comprehensively examined and compared optic nerve morphology, visual acuity, active axonal transport, and retinal glial reactivity in IL-6 deficient (*IL-6*-/-) and wildtype (WT) mice with 8 weeks of unilateral or bilateral microbead-induced glaucoma (microbead occlusion model). Together, our data indicate that IL-6 deficiency mitigates glaucoma-induced deficits in visual function and optic nerve structure without improvement in axon transport or reduction in microglia reactivity. This suggests that IL-6 may play specific role in the progression of RGC axonopathy from functional deficits to structural degeneration.

## Materials and methods

### Animals

Seven to nine month old male and female *IL-6*-/- mice (B6;129S2-*IL6*^*t*^^m1kopf^/J) and respective genomic controls (B6;129SF2/J) were used for all experiments. *IL-6*-/- mice contain a neomycin selection cassette in exon 2 of the IL-6 gene preventing transcription of the mRNA product (Kopf et al., 1994). Founder mice were obtained from Jackson Laboratories (Bar Harbor, ME) and experimental mice were bred and genotyped in-house using the following primers provided by Jackson Labs: 5′-TTC-CAT-CCA-GTT-GCC-TTC-TTG-G-3′, 5′-TTC-TCA-TTT-CCA-CGA-TTT-CCC-AG-3′ and 5′-CCG-GAG-AAC-CTG-CGT-GCA-ATC-C-3′. Mice were housed in accordance with NIH guidelines and maintained on a 12 h light/dark cycle with *ad libitum* access to standard mouse chow and water. This study was carried out in accordance with the ARVO statement for the use of animals in ophthalmic and vision research and was approved by the IACUC of Vanderbilt University Medical Center.

### Induction of ocular hypertension using the microbead occlusion model

Acute IOP elevation was induced in WT and *IL-6*-/- mice using the microbead occlusion model, as previously described (Sappington et al., 2010). For anterograde axonal transport, axon density measurements, and retinal gliosis, mice from both genotypes received a unilateral injection of 1.5 μl (1 × 10^6^ microbeads/mL) of 15 μm polystyrene beads conjugated to an Alexa Fluor 488 chromophore. The contralateral eye served as a surgical control and was injected with an equal volume of saline. For experiments looking at visual acuity and corneal integrity, 7–11 mice from both genotypes received bilateral injections of 1.5 μl microbeads and a separate cohort of mice served as controls and received bilateral injections of an equivalent volume of saline. All mice received two microbead/saline injections 4 weeks apart to raise IOP for a total of 8 weeks. Following IOP elevation, mice were sacrificed by transcardial perfusion of 50 ml of 1X PBS followed by 100 ml of 4% paraformaldehyde. Eye and brain tissue were stored in 4% PFA at 4°C until use.

### IOP measurements

IOP was measured in awake, behaving mice, using a Tonolab rebound tonometer (TonoLab; Reichert, Depew, NY), as previously described (Echevarria et al., 2013; Formichella et al., 2014; Echevarria et al., 2016). Prior to initial injection, mean baseline IOP for each mouse was calculated from approximately 60 individual readings taken over a period of 6 days (10 measurements/day) within a 2 week timeline. Following microbead or saline injections, weekly IOP was determined as the mean of 20–30 measurements, taken over 2–3 days (10 measurements/day) each week for a total of 8 weeks. IOP measurements were taken at the same time of day to remove any effect of circadian rhythm on IOP measurements. To avoid corneal irritation and discomfort, 0.5% proparacaine anesthetic drops (Akorn Inc, Lake Forest, IL), and lubricating eye drops were applied to each eye before and after IOP measurements were taken respectively.

### Immunohistochemistry

Immunohistochemistry of whole mount retinas was done as previously described (Sims et al., 2012; Echevarria et al., 2013, 2016). Primary antibodies against glial fibrillary acidic protein (GFAP, 1:500; Cat# Z033429-2; DAKO) to label astrocytes, ionized calcium-binding adapter molecule-1 (Iba-1, 1:250; Cat# 019-19741; WAKO) to label microglia, and β-Tubulin III (TUJ1, 1:500; Cat#845501; BioLegend) to label RGCs were used. Secondary antibodies were used at a concentration of 1:200 and consisted of donkey α-rabbit attached to either a Rhodamine Red-X (Cat# 711-295-152; Jackson Immuno Labs) or Alexa-647 (Cat# 711-605-152; Jackson Immuno Labs) fluorophore.

### Fluorescent *In-situ* hybridization

Generation of IL-6 probes and FISH in naïve WT and *IL-6*-/- whole mount retina were done as previously described (Crish et al., 2013). Probes were made against a nucleotide sequence encompassing exons 2–5 of *IL-6* [nucleotides 107–651 of (NCBI Ref Seq: NM_031168.2)]. The transcript inserted into the pGEM-T Easy Vector (Promega, Madison WI) was generated by PCR using primers to IL-6 (forward 5′-ATCCAGTTGCCTTCTTGGGACTGA-3′ and reverse 5′TGGCTAAGGACCAAGACCATCCAA-3′). Following FISH, retinas underwent immunohistochemistry as described above to label RGCs.

### Microscopy and image analysis

Imaging of whole mount retinas was done on an inverted confocal microscope (Olympus FV-1000; Center Valley, PA) through the Vanderbilt University Medical Center Cell Imaging Shared Resource Core. *IL-6* and β-Tubulin III labeling was imaged at 100X, while GFAP and Iba-1 was imaged at 60X. For both groups, 7–9 pseudo-random z-stack images in the mid central/mid-peripheral areas through the ganglion cell (GCL) and nerve fiber layers (NFL) of the retina were acquired using a digital camera and image analysis software (FV-100 ASW; Olympus). GFAP and Iba-1 percent area was calculated using NIS elements AR software (Nikon Instruments, Melville, NY), as previously described (Formichella et al., 2014). The area (mm^2^) of the image containing above background signal intensity of Iba-1 or GFAP (See **Figures 6A,B**; red labeling) was calculated and reported as a percentage of the total area of the image. Total area of each image and background signal threshold was equal among all images. Microglia cell density was calculated by counting the number of Iba-1 positive cell somas and dividing the counts by the area of the image.

### Anterograde axon transport measurements

Anterograde axonal transport capabilities of RGCs were assessed with cholera toxin beta-subunit (CTB) conjugated to a 488 fluorophore, as previously described (Crish et al., 2010; Formichella et al., 2014; Ward et al., 2014; Bond et al., 2016). Briefly, mice were given a 1.5–2 μl intravitreal injection of CTB (10 μg/μl in sterile ddH_2_O; Cat# C-34775, Life Technologies) using a 33 gauge needle attached to a Hamilton syringe under 2.5% isoflurane anesthesia. Five days after CTB injection, mice were sacrificed by transcardial perfusion as described above. To quantify axon transport, whole brains were cryopreserved in 30% sucrose for 24–48 h at 4°C. Using a sliding microtome, 50 μm sections were obtained through the superior colliculus (SC). CTB signal in these sections was imaged *en montage* at 10X, using a Nikon Eclipse T*i* inverted microscope (Nikon Instruments, Melville, NY). Anterograde axonal transport was quantified as previously described (Crish et al., 2010). Briefly, the SC from each image was outlined and CTB signal above background was divided by total pixel area to determine the volume of SC with CTB labeling. This value was used to create a colorimetric 2D retinotopic map of CTB transport ranging from 0% (blue) and 100% (red). Intact transport was defined as percent area with CTB signal >70% density (red/yellow).

### Axon density and nerve area

Axon density was measured in semi-thin sections of optic nerve as previously described (Sappington et al., 2010; Ward et al., 2014). Briefly, optic nerves were post fixed at least 48 h in 2.5% glutaraldehyde and embedded in epon. Semi-thin (700 nm) cross-sections of optic nerve near the chiasm were stained with 1% p-Phenylenediamine (PPD) and 1% toluidine blue to highlight myelin and glia, respectively. Optic nerve cross-sections were imaged *en montage* at 100X magnification on an upright Olympus Provis AX (Olympus, Melville, NY) microscope. To calculate axon density, a 50 × 50 μm grid mask was placed on the montaged image using NIS elements AR software. The number of axons was manually counted by a blind-observer in 8–10 squares of the grid. Each square counted was equal in area (0.0025 mm^2^). To measure nerve area, the circumference of the nerve was traced in montaged images of optic nerve cross-sections. Nerve area was calculated as the area (mm^2^) within this outline using NIS elements software.

### Neurobehavioral visual testing using optomotry

The optokinetic response is a naturally occurring reflex that serves as a functional tool for quantitative analyses of visual system function in mice (Douglas et al., 2005). Briefly, each mouse was placed on a platform surrounded by four LCD computer monitors. A sinusoidal grating of alternating white and black bars rotating in either a clock-wise or counter clock-wise fashion was projected on the monitors. Mice able to perceive the moving stimulus produced a reflexive movement of the head in the direction of the stimulus. The visual acuity of each mouse was measured by changing the spatial frequency of the black and white bars. The visual acuity threshold was determined as the highest spatial frequency for which reflexive tracking was noted. The presence of the reflexive head movement was recorded by an observer using a camera mounted above the mouse. Mice were tested for baseline visual acuity threshold 1–2 weeks before microbead/saline injection and 4 and 8 weeks post-initial microbead/saline injection.

### Corneal imaging using spectral domain optical coherence tomography (SD-OCT)

Mice were anesthetized with a ketamine/xylazine cocktail (80/5 μg/gram of mouse), pupils were dilated with 0.5% Tropicamide, and eyes kept moist with lubricating eye drops. Live volumetric scans of the cornea were obtained using SD-OCT running the Bioptogen ultra-high resolution spectral domain OCT system with cornea bore (Bioptogen, Morrisville, NC). Quantification of injury area was performed using Image J software (National Institute of Health).

#### Statistical analysis

Statistical analysis was conducted with SigmaPlot Version 11.1 (Systat Software Inc, San Jose, CA). For baseline and delta baseline IOP comparisons between WT and *IL-6*-/-, a Mann-Whitney Rank Sum test and a One-Way ANOVA with Holm-Sidak *post-hoc* correction was done respectively. For post injection IOP comparisons, a One-Way ANOVA on RANKS with Dunn's *post-ho*c correction was done. For corneal wound area measurements, a two-tailed *t*-test was done between WT and *IL-6*-/- mice at each time point. Differences in visual acuity throughout the 8 week experimental time course were assessed with a One-Way Repeated Measures ANOVA between baseline visual acuity, acuity at 4 weeks post initial injection, and 8 weeks post initial injection within each experimental group. Differences between all experimental groups at each time point were assessed with a One-Way ANOVA with Holm-Sidak *post-hoc* correction. Differences in percent baseline visual acuity at 8 weeks between all experimental groups were assessed with a One-Way ANOVA on RANKS with Dunn's *post-hoc* correction. All other comparisons were made with a One-Way ANOVA on RANKS with Dunn's *post-hoc* correction (percent glia coverage, microglia cell density) or a One-Way ANOVA with Holm-Sidak *post-hoc* correction (SC transport, axon density/nerve area). For all, *p* < 0.05 were considered statistically significant.

## Results

### IL-6 deficiency does not affect microbead-induced elevations in IOP

**Figure 1:**
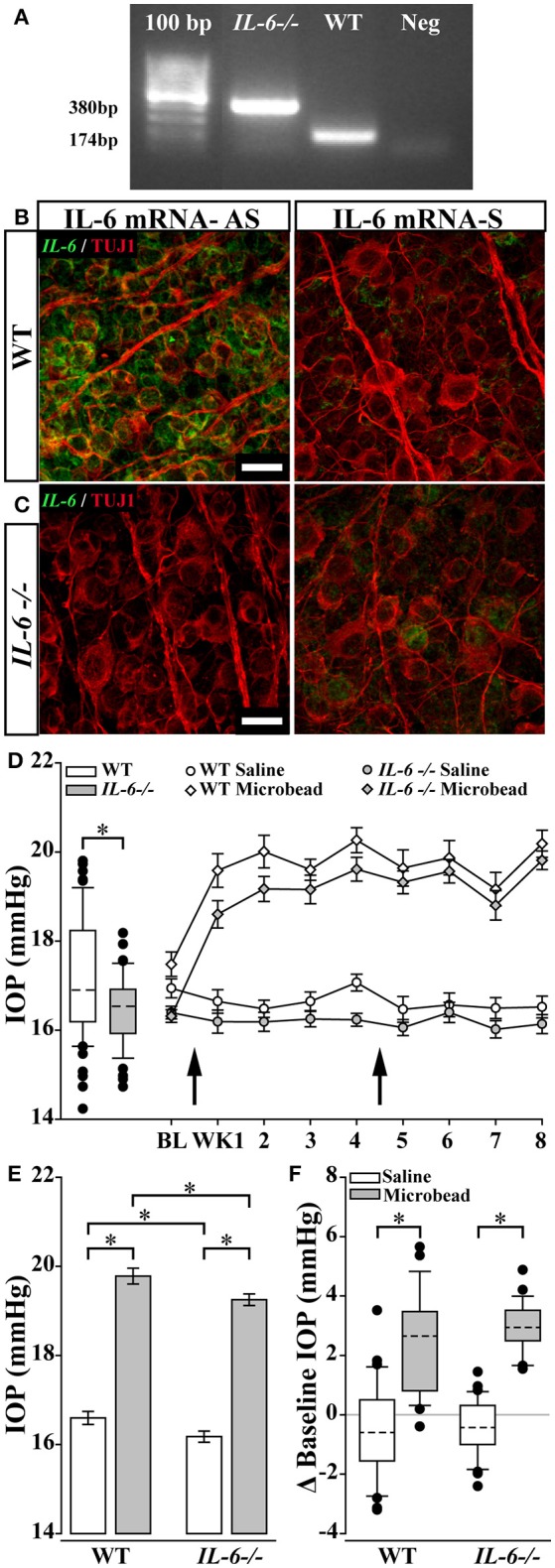
IL-6 deficiency does not affect magnitude and duration of microbead induced ocular hypertension. **(A)** Representative gel from a PCR confirming the *IL-6*-/- genotype. PCR amplification of exon 2 leads to a PCR product of 380 bp in *IL-6*-/- mice and a 174 bp PCR product in WT mice. No band present in negative control lane. Products were run along a 100 bp ladder. **(B,C)**. Representative 100X confocal image of retinal whole mount from WT **(B)** and *IL-6*-/- mice **(C)** incubated with an anti-sense (AS; left) or sense probe (S; right) against *IL-6* mRNA (right). Signal from AS probe (**B**, green) is found within β-Tubulin (TUJ1) positive RGCs (red) in WT mice **(B)**. No significant signal was present in retinas from *IL-6*-/- mice (**C**; left) or during incubation with sense probe (**B,C**; right). Scale bars = 20 μm. **(D;** left) Boxplot of baseline IOP of WT (white) and *IL-6*-/- (gray) mice from all experimental cohorts prior to microbead/saline injection. Baseline IOP of *IL-6*-/- mice is decreased by 4% compared to baseline IOP of WT mice. (**D**; right) Line plot (mean ± SEM) showing baseline and weekly post saline (circle) or microbead (diamond) IOP in WT (white) or *IL-6*-/- (gray) eyes. Arrows indicate time of saline/microbead injections. Throughout the 8 week experiment, microbead injected eyes from both WT and *IL-6*-/- show a 15–20% increase in IOP compared to baseline measurements and saline injected eyes. **(E)** Bar graph of average IOPs (mean ± SEM) taken post initial microbead (gray) or saline (white) injection in both WT and *IL-6*-/- mice. A significant IOP increase in microbead- injected eyes vs. saline- injected eyes is seen in both genotypes. A genotype specific IOP reduction is seen in both saline- and microbead- injected *IL-6*-/- mice. **(F)** Boxplot showing magnitude of IOP difference in saline (white) and microbead (gray) injected WT and *IL-6*-/- mice compared to baseline measurements. A significant elevation in IOP is present in microbead- injected eyes compared to saline- injected eyes in both genotypes. However, no genotype specific differences in IOP seen. ^*^p < 0.05. *n* = 26–34 eyes/genotype/condition. Dashed lines in box plot indicate median value.

To confirm genetic ablation of IL-6, we conducted PCR to confirm the presence of the neomycin selection cassette in exon 2 of the *IL-6* gene. In all *IL-6*-/- mice used in this study, PCR amplification of exon 2 revealed a 380 bp product in the *IL-6*-/- mouse compared to the 174 bp PCR product in the WT mouse (Figure 1A). Loss of *IL-6* mRNA was corroborated using *in situ* hybridization. In a subset of WT and *IL-6*-/- whole mount retina co-immunolabeled with the RGC-specific marker β-Tubulin (TUJ1), labeling for *IL-6* mRNA using an antisense fluorescent *in situ* hybridization (FISH) probe showed robust signal that co-localized to TUJ1+ positive RGCs in WT mice (Figure 1B; left). Conversely, anti-sense labeling for *IL-6* mRNA was not detected in *IL-6*-/- mice (Figure 1C; left). Similarly, significant *IL-6* mRNA labeling was not detected following incubation with the sense probe in either genotype (Figures 1B,C; right). To examine the impact of IL-6 deficiency on the progression of IOP-induced RGC neurodegeneration, we utilized the microbead occlusion model (Sappington et al., 2010) of glaucoma to elevate IOP for a total of 8 weeks in WT and *IL-6*-/- mice. Baseline IOP was 4% lower in *IL-6*-/- mice (16.4 +/− 0.79 mmHg), compared to WT mice (17.1 +/− 1.32 mmHg; *p* < 0.01; Figure 1D; left). Microbead injection increased IOP by ~ 20%, as compared to saline-injected controls for both genotypes (WT; *p* < 0.001, *IL-6*-/-; *p* < 0.001, Figure 1D; right and Figure 1E). In accordance with baseline IOP measurements, the mean IOP (mmHg) for both saline- (*p* < 0.05) and microbead-injected (*p* < 0.05) was lower in *IL-6*-/- mice than their WT counterparts (Figure 1E). However, with respect to baseline IOP, the magnitude of IOP elevation was similar (~2.5 mmHg) in microbead-injected WT and *IL-6*-/- mice (*p* > 0.05, Figure 1F).

### IL-6 deficiency preserves optic nerve structure following IOP elevation

**Figure 2:**
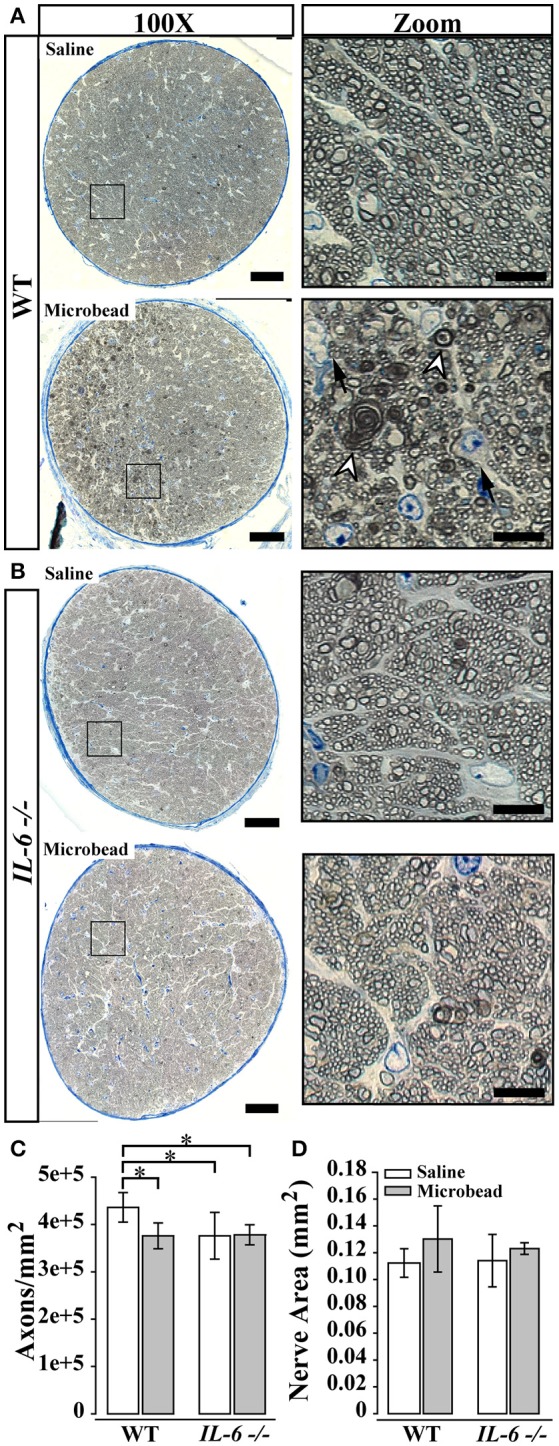
IL-6 deficiency mitigates axon degeneration caused by IOP elevation. **(A,B)** Representative 100X montaged optic nerve cross sections from WT **(A)** and *IL-6*-/- **(B)** optic nerves following saline (top) or microbead (bottom) injection. Black box in montaged image (left) corresponds to location of zoomed images highlighting axon and glia (right). IOP elevation results in increased glial infiltration (black arrows) and degenerative axon profiles (white arrow heads) in optic nerves from microbead injected WT, but not *IL-6*-/- mice. **(C)** Bar graph of average (mean ± STDEV) myelinated axon density measurements in WT and *IL-6*-/- mice following saline (white) or microbead (gray) injection. Saline- injected *IL-6*-/- mice show a genotype specific decrease in myelinated axon density compared to saline- injected WT mice. However, microbead- injected WT eyes show a significant 15% decrease in myelinated axon density compared to saline- injected WT eyes, while no difference is seen between microbead- and saline- injected *IL-6*-/- eyes. **(D)** Bar graph of average nerve area (mean ± STDEV) among groups shows no significant difference. ^*^p < 0.05. *n* = 40–50 density measurements/genotype/group. Scale bars = 50 μm for 100X montaged optic nerves and 10 μm for zoomed images.

In glaucoma, degeneration of the optic nerve starts at the distal end of the optic nerve and progresses in a distal to proximal fashion (Crish et al., 2010). Unlike the distal optic nerve of saline-injected WT mice (Figure 2A; top), the distal optic nerve of microbead-injected WT mice presented with signs of structural pathology, including increased glial infiltration and degenerating axon profiles (Figure 2A; bottom). This was accompanied by a slight enlargement in nerve area (Figure 2C; left) and a 15% decrease in axon density, as compared to saline-injected mice (*p* < 0.05, Figure 2D; left). In contrast, while distal optic nerves from microbead-injected *IL-6*-/- mice presented with some gliosis, no change in degenerating axon profiles were noted (Figure 2B). Similarly, there were no measurable changes either in nerve area (Figure 2C; right) or myelinated axon density (*p* > 0.05, Figure 2D; right), as compared to saline-injected *IL-6*-/- mice. However, independent of IOP, optic nerves from *IL-6*-/- mice contained approximately ~15% fewer myelinated RGC axons than those from WT mice (*p* < 0.05, Figure 2D).

### IL-6 deficiency does not prevent IOP-induced deficits in anterograde axon transport

**Figure 3:**
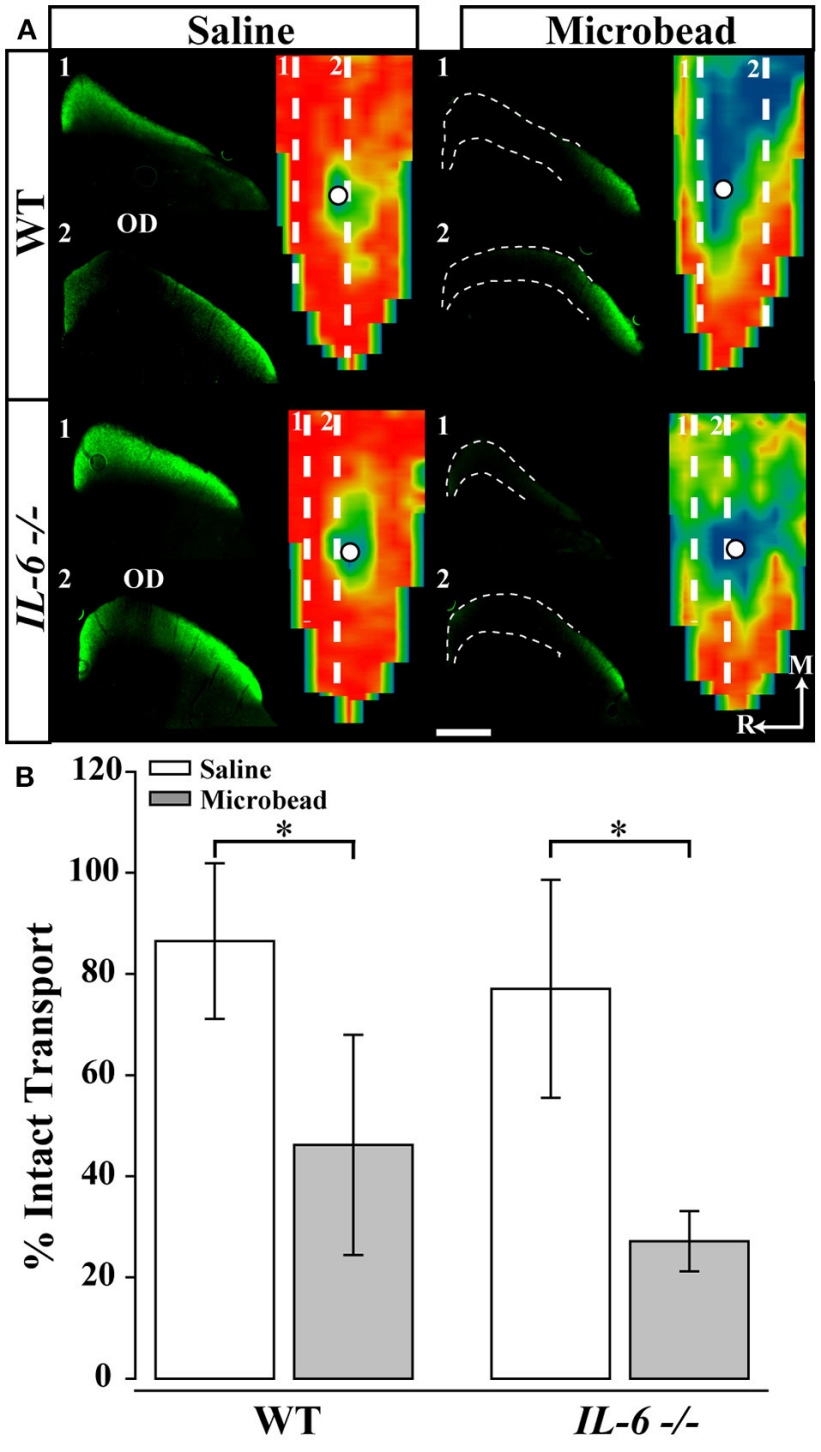
IL-6 deficiency does not alter IOP-dependent deficits in anterograde axon transport. **(A)** Representative coronal sections through the superior colliculus (SC) and respective retinotopic heat maps after 5 days of CTB transport in WT (top) and (bottom) *IL-6*-/- mice. Outlines in coronal sections indicate areas of transport deficits. Dashed lines in map indicate position of coronal section. Density of the CTB signal for heat maps range from 0% (blue) to 50% (green) to 70% (yellow) to 100% (red). Numbered, dashed lines in retinotopic maps indicate the location of respective coronal section and white circles indicate position of the optic disk (OD). Medial (M) and rostral (R) orientations are indicated. **(B)** Bar graph showing average percent intact transport (mean ± STDEV, >70% density of CTB signal; red/yellow areas) from SC following saline (white) or microbead (gray) injection in WT (left) and *IL-6*-/- (right) mice. SC from both microbead- injected WT and *IL-6*-/- mice show a ~50% deficit in intact axon transport compared to saline- injected mice. ^*^p < 0.05. *n* = 5–6 SC/genotype/condition. Scale bars = 500 μm for all images.

Previous reports indicate that functional deficits in anterograde axon transport along the optic projection precede structural degeneration of RGC axons in glaucoma (Crish et al., 2010, 2013). To measure active anterograde transport in RGC axons, we injected the active uptake, active transport tracer cholera toxin beta subunit (CTB) into the vitreous of *IL-6*-/- and WT mice 8 weeks after the initial microbead or saline injection. We measured anterograde transport of CTB from RGC soma in the retina to RGC terminals in the superior colliculus (SC) by quantifying CTB labeling in serial sections of SC and generating 2D reconstructions of CTB labeling in the SC (Figure 3A). In WT mice, 8 weeks of elevated IOP led to a ~50% decrease in CTB transport to the SC, as compared to saline-injected mice (*p* < 0.001, Figure 3A; top, Figure 3B; left). Interestingly, in *IL-6*-/- mice, IOP elevation also resulted in a ~50% decrease in CTB transport (*p* < 0.001, Figure 3A; bottom, Figure 3B; right). Similar to previously published studies (Crish et al., 2010; Lambert et al., 2011; Ward et al., 2014), these deficits occurred in a sectoral manner, extending from the periphery toward the optic disc in WT and *IL-6*-/- mice (Figure 3A). No differences in axon transport were noted between genotype in saline-injected animals (*p* > 0.05, Figure 3B).

### IL-6 deficiency preserves visual acuity following IOP elevation

**Figure 4:**
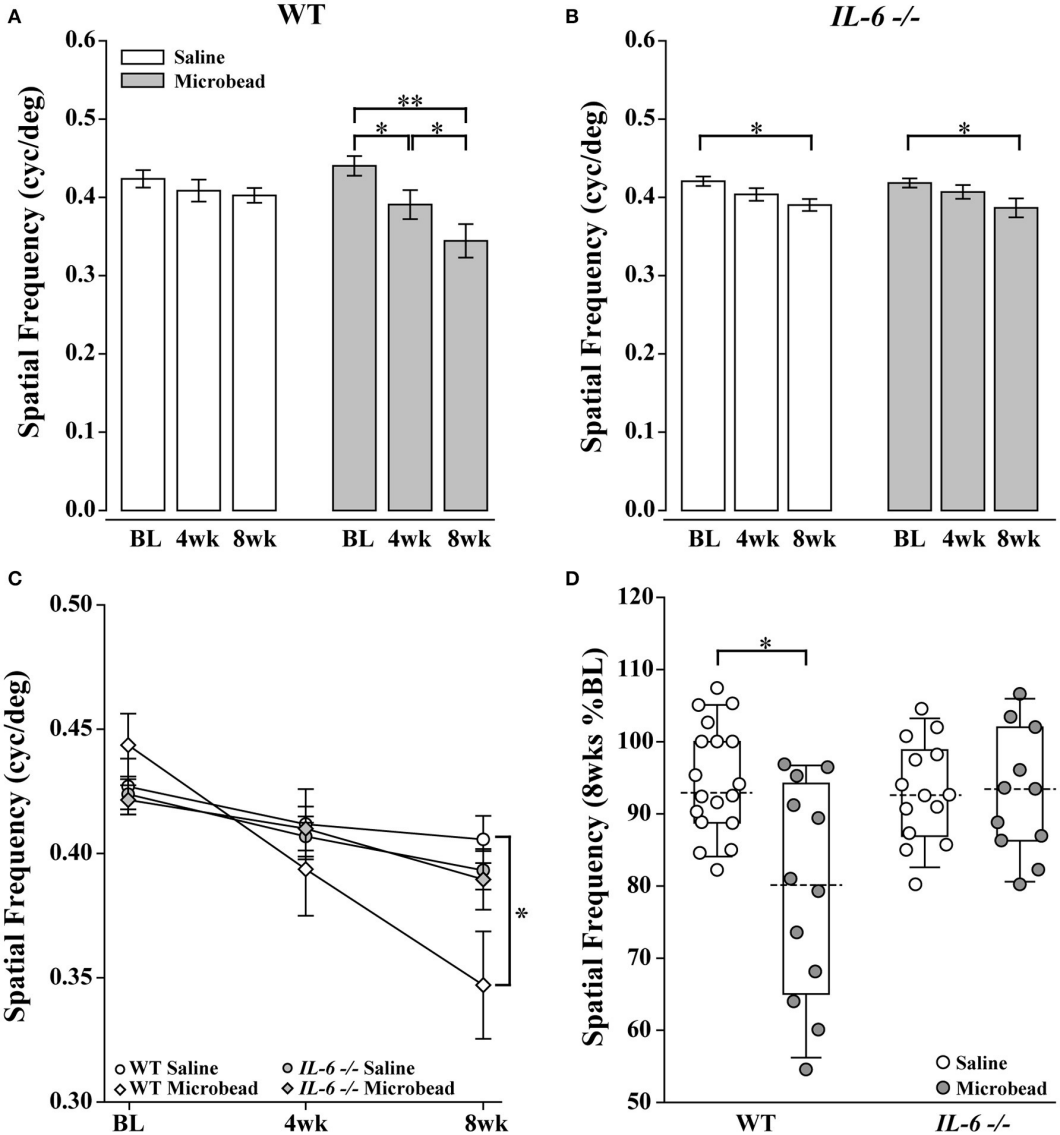
*IL-6*-/- mice are resistant to IOP-induced deficits in visual acuity. **(A)** Bar graph showing average visual acuity threshold (mean ± SEM) of WT mice at baseline and 4 and 8 weeks post initial saline (white) or microbead (gray) injection. WT mice injected with microbeads show a significant decrease in visual acuity at both 4 weeks and 8 weeks post-injection compared to baseline. **(B)** Bar graph showing average visual acuity threshold (mean ± SEM) of *IL-6*-/- mice at baseline and 4 and 8 weeks post initial saline (white) or microbead (gray) injection. *IL-6*-/- mice injected with either saline or microbeads show a significant decrease in visual acuity at 8 weeks compared to baseline. **(C)** Line graph comparing visual acuity of WT saline (white circle), WT microbead (white diamond), *IL-6*-/- saline (gray circle) and *IL-6*-/- microbead (gray diamond) at each time point. Visual acuity decreases significantly in microbead-injected WT mice compared to saline-injected WT mice. Visual acuity does not differ between saline- and microbead- injected *IL-6*-/- or between genotypes. **(D)** Boxplot of the percent visual acuity remaining at 8 weeks compared to baseline measurements for WT and *IL-6*-/-. WT mice injected with microbeads show a significant decrease in the remaining visual acuity when compared to the saline- injected WT mice. *IL-6*-/- mice injected with microbeads show no difference in the remaining visual acuity when compared to the saline injected *IL-6*-/- cohort. ^*^p < 0.05, ^**^p < 0.001. *n* = 13–17/group. Dashed lines in boxplot indicate median value of data set.

Loss of vision in glaucoma is irreversible and caused by degeneration of RGCs and their axons (Calkins, 2012). To ensure detection of any vision loss associated with microbead-induced glaucoma, we performed bilateral injections of microbeads in one cohort of WT and *IL-6*-/- mice. A second cohort received bilateral injections of saline. We measured visual acuity by optomotry every 4 weeks for the duration of the experiment. Over the course of 8 weeks, microbead-injected WT mice exhibited significant depreciation of visual acuity at each time point compared to baseline, resulting in an overall 22% decrease in visual acuity (*p* < 0.001 for all, Figure 4A; gray). However, in saline-injected WT mice, visual acuity did not significantly differ from baseline at either time point (*p* > 0.05, Figure 4A; white). Comparison of visual acuity between saline- and microbead-injected WT mice revealed a significant ~15% decrease in both raw visual acuity (*p* < 0.05, Figure 4C) and percent baseline visual acuity (*p* < 0.05, Figure 4D). In *IL-6*-/- mice, visual acuity dropped 8% with either saline (*p* < 0.01) or microbeads (*p* < 0.05), as compared to baseline acuity (Figure 4B). However, this initial reduction in visual acuity did not differ between saline- and microbead-injected *IL-6*-/- mice (*p* > 0.05, Figures 4C,D) and remained unchanged between 4 and 8 weeks for both groups (saline: *p* > 0.05; microbead: *p* > 0.05; Figure 4B). That this slight decrease in visual acuity was noted in both saline- and microbead-injected *IL-6*-/- mice and remained stable for the 8 week experiments suggest that it arises from an IOP-independent influence. No difference in visual acuity was noted between WT and *IL-6*-/- mice at any time point (*p* > 0.05, Figure 4C).

### *IL-6*-/- mice exhibit deficits in corneal wound healing

**Figure 5:**
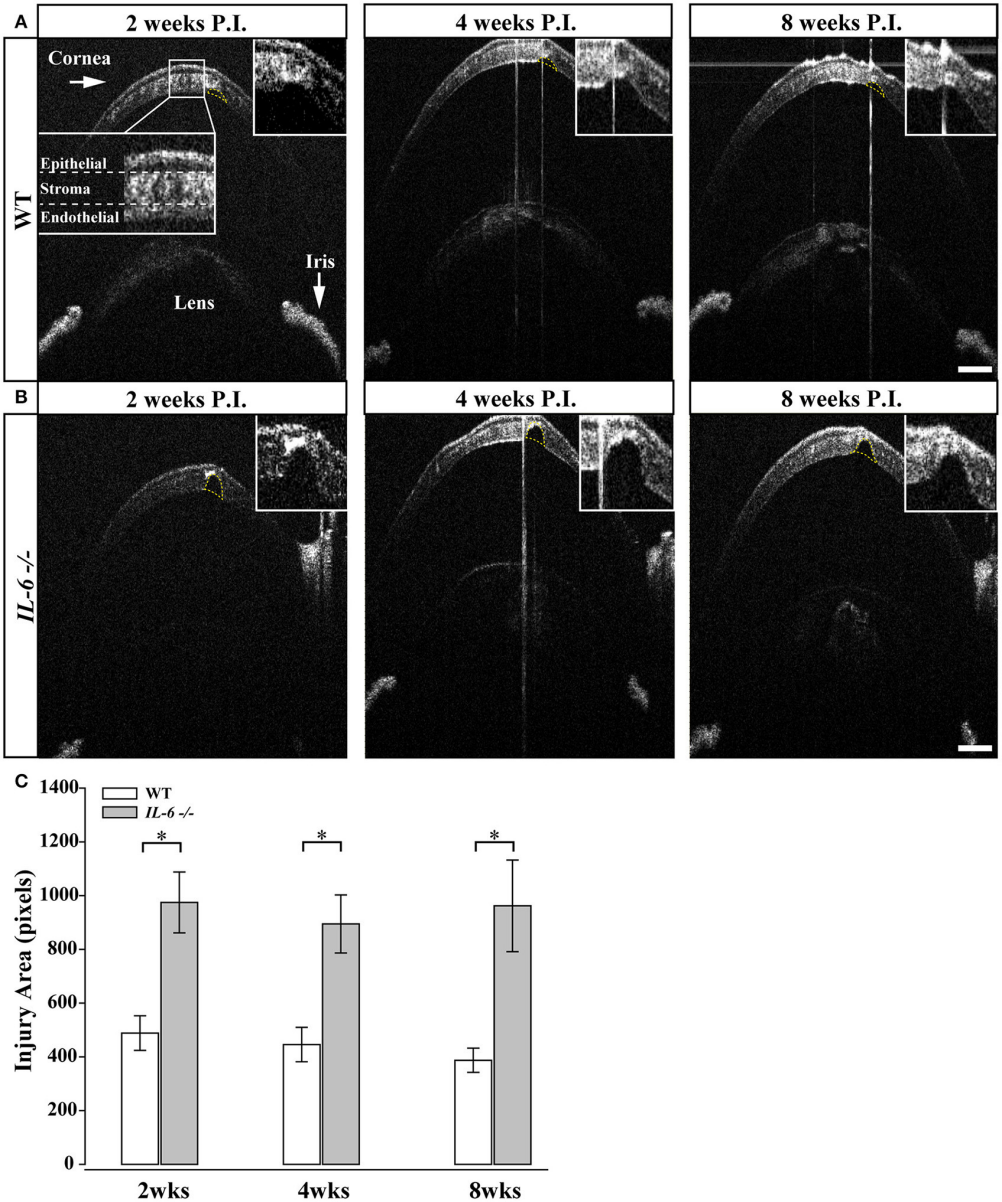
*IL-6*-/- mice present with defects in corneal wound healing following microbead/saline injection.**(A)** Representative images of corneal wounds at 2, 4 and 8 weeks post initial injection in WT mice. Insert (**A;** far left) outlines layers of the cornea. 2 weeks after corneal puncture due to saline or microbead delivery, WT mice left display small gaps in the corneal stroma and endothelium (yellow dotted lines). The size of the injury persists after 4 (middle) and 8 (right) weeks after injury. **(B)** Representative images of corneal wounds at 2, 4, and 8 weeks post injection in *IL-6*-/- mice. *IL-6*-/- mice however, present with significantly larger gaps 2 weeks (left) in the corneal stroma after puncture that also persists at 4 (middle) and 8 (right) weeks after injury. **(C)** Bar graph showing quantification of corneal injury area (mean ± SEM). *IL-6*-/- mice have significantly larger corneal injuries at all time points. Size of the wound area did not change significantly over time in either WT or *IL-6*-/-. ^*^p < 0.05. *n* = 9 eyes/genotype/group. Scale bars= 100 μm for all images.

Previous studies indicate *IL-6*-/- mice exhibit deficits in wound healing (Lin et al., 2003; McFarland-Mancini et al., 2010). As the microbead/saline injections require puncturing of the cornea, we used spectral-domain optical coherence tomography (SD-OCT) imaging to examine whether perturbed healing of the cornea could underlie the reduction in visual acuity noted in both saline and microbead-injected *IL-6*-/- mice. Two weeks following intra-cameral injection of saline or microbeads in WT eyes, SD-OCT imaging revealed complete closure of the epithelial layer and approximately 2/3 closure of the stroma and endothelial layers at the injection site (Figure 5A). Quantification of the remaining corneal wound revealed no significant change over the remaining 6 weeks (*p* > 0.05; Figure 5C). In *IL-6*-/- mice, SD-OCT imaging revealed complete closure of the epithelial layer by 2 weeks. However, limited closure of the stroma and endothelial layers was noted (Figure 5B). This reduction in stroma and endothelial wound healing was noted throughout the 8 week experiment (Figure 5B). Quantification of corneal injury revealed that the area of corneal wound was 2-fold larger in *IL-6*-/- mice than in WT mice at all three time points (*p* < 0.05; Figure 5C). Like WT mice, the area of the corneal wound did not change over the 8 week experiment in *IL-6*-/- (*p* > 0.05; Figure 5C).

### IL-6 deficiency enlarges the microglia population in retina

**Figure 6:**
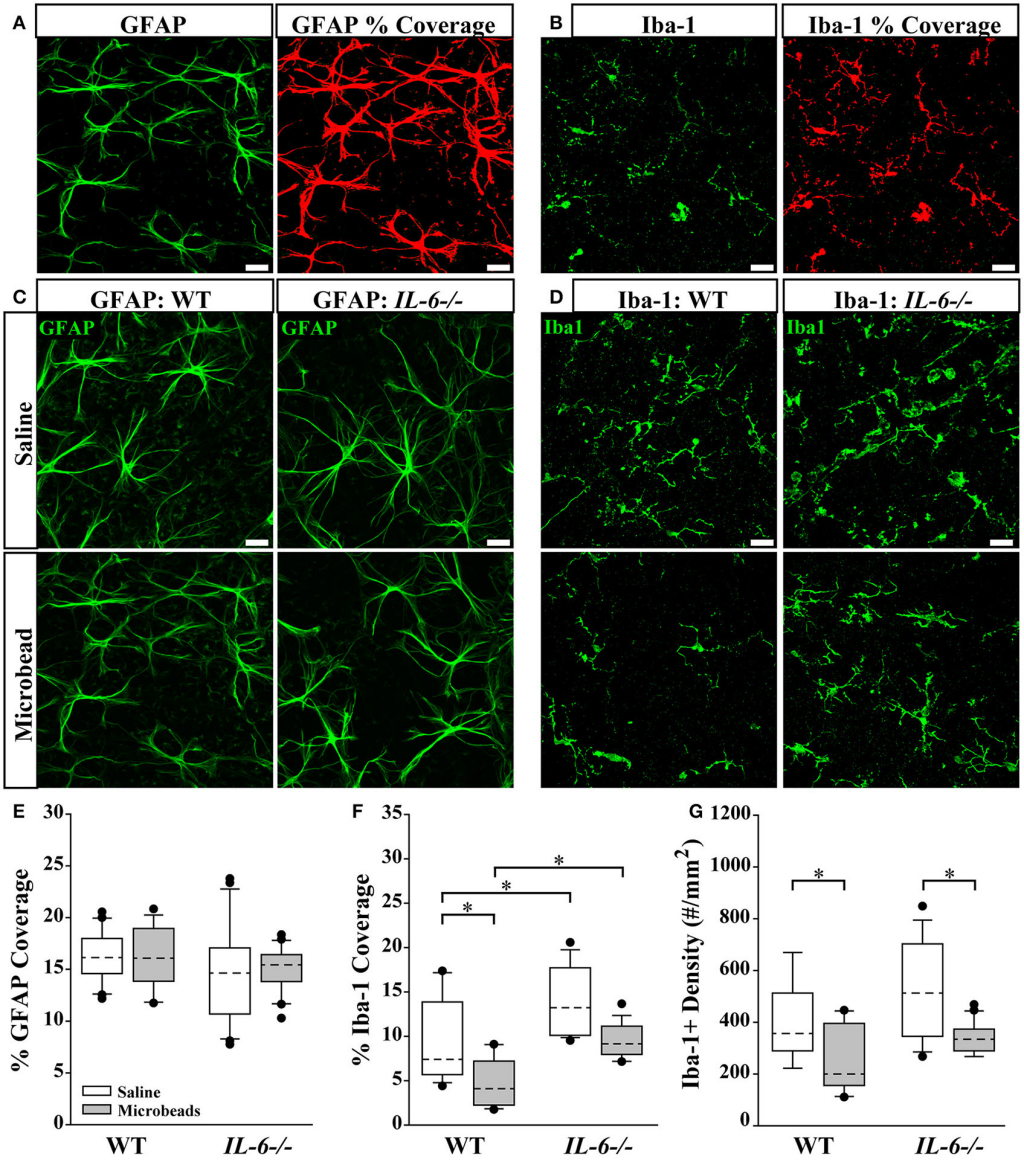
IL-6 deficiency affects microglial coverage regardless of IOP. **(A,B)** Representative 60X confocal images depicting quantification of percent coverage of GFAP+ astrocytes (green; **A**, left) and Iba-1+ microglia (green; **B**, left). The area containing above background signal for GFAP and Iba-1 was highlighted in red (**A,B**; right) and divided against the total area of the image to obtain the percent coverage measurements. **(C,D)** Representative 60X confocal images depicting GFAP+ astrocyte (green; **C**) and Iba-1+ microglia (green; **D**) labeling in whole mount retina of microbead- and saline- injected WT and *IL-6*-/- eyes. **(E)** Boxplot of percent coverage of GFAP+ astrocytes in saline (white) and microbead (gray) injected WT (left) and *IL-6*-/- (right) eyes. No genotype or IOP dependent changes were calculated. **(F)** Boxplot of percent coverage of Iba-1+ microglia in saline- (white) and microbead- (gray) injected WT (left) and *IL-6*-/- (right) eyes. While an IL-6 dependent increase in percent coverage of microglia is seen in both saline and microbead- injected eyes, only an IOP dependent decrease is seen in microbead- injected WT eyes. **(G)** Boxplot of Iba-1+ microglia cell density (counts/mm^2^) in saline- (white) and microbead- (gray) injected WT (left) and *IL-6*-/- (right) eyes. IOP dependent decreases in microglia counts are evident in microbead- injected eyes are evident in both WT and *IL-6*-/- eyes. ^*^p < 0.05. *n* = 6–8 images/eye/condition/genotype. Scale bars= 30 μm for all images. Dashed lines in boxplot indicate median value of data set.

Recent studies suggest that changes in glial reactivity in the retina occur in response to IOP elevation, and impact RGC degeneration in both genomic and inducible models of glaucoma (Martin et al., 2003; Sappington and Calkins, 2006; Inman and Horner, 2007; Bosco et al., 2008; Johnson and Morrison, 2009; Johnson et al., 2011; Echevarria et al., 2013; Lye-Barthel et al., 2013; Formichella et al., 2014; Hines-Beard et al., 2016). To determine whether IL-6 deficiency alters glial reactivity associated with RGC axonopathy, we performed a morphological analysis of astrocyte and microglia reactivity in retina. We visualized astrocytes and microglia in whole-mount retina from saline- and microbead- injected WT and *IL-6*-/- mice with immunolabeling against the astrocyte- specific label glial fibrillary acidic protein (GFAP) and the microglia- specific marker ionized calcium binding adaptor molecule (Iba-1). While Iba-1 labels various types of myeloid cells, we selected this marker because (1) 100% of retinal microglia are Iba-1 positive (Bosco et al., 2011), (2) Iba-1 expression remains rather stable across activation states compared to other microglia/macrophage markers (Bosco et al., 2011) and (3) with the exception of amoeboid microglia, other myeloid cells and microglia can be readily distinguished by morphology. To account for changes in both glia density and hypertrophy/ramification, we quantified the percent of retinal area covered by GFAP+ astrocytes (Figure 6A) and Iba-1+ microglia (Figure 6B). Our previous work indicates that percent area coverage is a highly reliable measure of reactivity (Formichella et al., 2014). GFAP immunolabeling revealed no gross genotype- or IOP-dependent changes in astrocytic morphology (Figure 6C). Quantification of astrocyte coverage confirmed no significant change in astrocyte morphology with respect to either IOP or genotype (*p* > 0.05 for all; Figure 6E). In contrast, Iba-1 immunolabeling revealed qualitative changes in microglia that appeared to relate to both genotype and IOP (Figure 6D). Quantification revealed 32% more microglia coverage in saline-injected *IL-6*-/- mice vs. WT mice (*p* < 0.05; Figure 6F). IOP elevation decreased microglia coverage by 45% in WT retina (*p* < 0.05; Figure 6F), as compared to saline-injected controls (Figure 6F). While it appeared as if there was decreased microglial coverage in *IL-6*-/- retina following IOP elevation compared to saline-injected controls, it did not reach statistical significance (*p* > 0.05; Figure 6F). Additionally, microglia coverage remained higher in *IL-6*-/- mice than in WT mice following IOP elevation (*p* < 0.05, Figure 6F). Based on qualitative assessment, IOP-induced changes in percent area coverage appeared to arise from changes in microglia density (Figure 6D). To quantitatively test this observation, we measured the density of Iba-1+ microglia across all experimental groups. We found that microbead-induced IOP elevation decreased the density of microglia by 37% in WT retina (*p* < 0.05) and by 36% in *IL-6*-/- mice (*p* < 0.05), as compared to saline-injected controls (Figure 6G). There was no significant difference in the density of microglia between WT and *IL-6*-/- mice regardless of treatment (*p* > 0.05; Figure 6G).

## Discussion

The present work investigated the relevance of IL-6 signaling to RGC axonopathy following microbead-induced IOP elevation. By comparing functional and structural outcomes of RGC degeneration in *IL-6*-/- and WT mice, we were able to link IL-6 signaling with specific events in RGC axonopathy. These studies delineate a role for IL-6 in the progression from functional deficits to structural degeneration within the axonopathy continuum. Secondarily, our data also indicated a role for IL-6 in corneal wound healing and potentially, IOP regulation.

Glaucoma is associated with elevated IOP. Current therapies, directed toward lowering IOP, delay pathology (Calkins, 2012). Not surprisingly, animal models of glaucoma are generally characterized by elevated IOP and subsequent degeneration of RGCs. Despite modest differences in baseline IOP, the magnitude of IOP elevation achieved with microbead occlusion was identical in WT and *IL-6*-/- mice (Figure 1). This indicates that IL-6 deficiency does not impact efficacy of IOP elevation in this model. Intracameral injection of saline and microbeads requires a small diameter (approx. 100 μm) puncture in the cornea. OCT imaging revealed that IL-6 deficiency resulted in a larger corneal wound and impeded stitching and filling of the stromal and endothelial layers of the cornea, which was visible in WT mice within 2 weeks of puncture (Figure 5). Interestingly, the epithelial layer of the cornea in *IL-6*-/- mice was indistinguishable from WT mice (Figure 5). This suggests that IL-6 signaling plays a prominent role in healing of stromal and endothelial, but not epithelial, layers of cornea. That a deficit in corneal wound healing was noted in our studies is not surprising, as IL-6 is strongly associated with wound healing and tissue regeneration in other systems (Blindenbacher et al., 2003; Lin et al., 2003; Tiberio et al., 2008; McFarland-Mancini et al., 2010).

Structural degeneration of the optic nerve and vision loss are the two hallmarks of glaucoma. In our study, IL-6 deficiency preserved both the structure of RGC axons and visual acuity following 8 weeks of elevated IOP. Consistent with previous findings (Sappington et al., 2010; Lambert et al., 2011; Ward et al., 2014; Bond et al., 2016), microbead-induced IOP elevation decreased axon density, while increasing degenerative axon profiles and macrogliosis in WT optic nerves (Figure 2). This was accompanied by a significant and IOP-dependent decrease in visual acuity (Figure 4). In contrast, *IL-6*-/- mice exhibited no *IOP-dependent* changes in visual acuity (Figure 4) or optic nerve outcomes, including axon density, degenerative axon profiles or macrogliosis (Figure 2). However, visual acuity decreased modestly, but significantly (8%), in an *IOP-independent* manner following both saline and microbead injection in *IL-6*-/- mice (Figure 4). That this decrease was noted following both types of injection and did not change over time, it is highly likely that visual acuity was negatively impacted by the observed deficits in corneal wound healing (Figure 5). Together, these data indicate that IL-6 signaling impacts RGC axonopathy prior to the onset of both structural degeneration and decreased visual function.

Previous studies indicate that RGC axonopathy in glaucoma progresses in a distal to proximal fashion and that deficits in active, anterograde transport precede structural degeneration of the optic nerve (Crish et al., 2010, 2013). Consistent with previously published studies (Sappington et al., 2010; Lambert et al., 2011; Ward et al., 2014; Bond et al., 2016), microbead-induced IOP elevation in WT mice resulted in a 50% decrease in anterograde transport of CTB to the SC (Figure 3). Interestingly, *IL-6*-/- mice exhibited a similar decrease in anterograde transport (Figure 3). This suggests that IL-6 signaling does not play a significant role in the development of axon pathology that leads to deficits in axon transport. Together with the optic nerve and visual function analyses, these data delineate a temporal window in which IL-6 signaling contributes to RGC axonopathy. Specifically, this temporal window begins after the onset of axon transport deficits and prior to the onset of structural degeneration and decreased visual function. More generally, our data suggest that axon transport deficits and structural degeneration of axons occurs via, at least partially, independent mechanisms.

Given that IL-6 is typically associated with inflammatory functions, we assessed microglia and astrocyte reactivity in the GCL and NFL. Not surprisingly, our analysis revealed a strong association between IOP, IL-6, and microglia, the “resident” immune cell of the CNS. Elevated IOP decreased microglia coverage in the GCL/NFL of WT mice (Figure 6). This was attributable to a decrease in the density of microglia (Figure 6). Interestingly, elevated IOP also decreased the density of microglia in *IL-6*-/- mice, but did not sufficiently reduce microglia coverage to a statistically significant level (Figure 6). Microglia coverage was greater in *IL-6*-/- than in WT mice, regardless of IOP (Figure 6). This IL-6-dependent increase in microglia coverage was not attributable to changes in microglia density, suggesting that it likely arises from changes in size and extent of ramification. Based on previous literature, changes in microglia density likely arise from migration of microglia to other retinal layers, particularly the outer retina (Rojas et al., 2014). These findings suggest that IL-6 may be more relevant for microglia ramification/activation state than microglia migration or temporal onset of microglia reactivity in this model. Unlike microglia, astrocyte reactivity did not appear to associate with IOP elevation or IL-6 deficiency (Figure 6). The former is contrary to studies of astrocyte reactivity in other glaucoma models, where both retinal astrocyte hypertrophy and hypotrophy are associated with elevated IOP. Notably, most of these studies were conducted in either chronic models (Inman and Horner, 2007; Formichella et al., 2014) or inducible models with much higher IOP elevation (Wang et al., 2000; Gallego et al., 2012). Differences in the duration and magnitude of IOP elevation, as well as severity of RGC pathology, could account for our contradictory findings. Together, these data suggest that IL-6 signaling generally associates with microglia rather than astrocytes. This is supported by our previous work indicating that retinal microglia, but not retinal astrocytes, induce IL-6 expression in response to elevated pressure (Sappington and Calkins, 2006, 2008; Sappington et al., 2006).

While our findings support a role for IL-6 in the progression RGC degeneration in glaucoma, we utilized *IL-6*-/- mice that are generated from homozygous pairing. As such, these mice are deficient in IL-6 throughout development as well as in adulthood. In this case, we are unable to differentiate IL-6-dependent outcomes arising from IL-6 signaling during disease and those arising from developmental IL-6 signaling. Our findings indicate that there are at least two developmental ramifications pertinent to our investigation:

First, IL-6 deficiency modestly, but significantly, decreased baseline IOP by ~4% compared to WT (Figure 1). There is some indication that modulation of IL-6 signaling accompanies IOP elevations in human patients, including both primary open angle and angle closure glaucoma (Takai et al., 2012; Engel et al., 2014; Huang et al., 2014; Du et al., 2016). However, in a recent study of porcine anterior segment, IL-6 did not appear to alter outflow facility that determines IOP (Birke et al., 2011). Our data suggest that, at least in mice, IL-6 signaling may impact the IOP “set-point” either through modulation of aqueous dynamics or during development of the anterior chamber. That the relative increase in IOP achieved with microbeads was equivalent in WT and *IL-6*-/- mice indicates the latter may be more relevant. Most importantly, the magnitude of change in IOP, rather than absolute IOP, appears to be of greater import for disease etiology in both animal models and human patients. In humans, IOP fluctuations, defined as the difference between the highest and lowest IOP, is greater in glaucoma patients than non-glaucoma patients (Nouri-Mahdavi et al., 2004; Sihota et al., 2005; De Moraes et al., 2011; Tan et al., 2017; Tojo et al., 2017) and is predictive of glaucoma progression (Nouri-Mahdavi et al., 2004; Sihota et al., 2005). Likewise, the magnitude of peak IOP measurement predicts progression in glaucoma patients, even for those with IOP successfully lowered by conventional treatments (Nouri-Mahdavi et al., 2004). In animal models, IOP elevations are often presented as a change in cumulative IOP, which documents the change in IOP from control eyes or baseline over time. This measure is well-documented as a strong predictor of RGC pathology (Gao and Jakobs, 2016). Additional studies of strain differences indicate that absolute IOP does not necessarily correlate with RGC pathology (Cone et al., 2010, 2012). In these studies, some strains present with less severity despite higher IOP elevations on the order of several mmHg (Cone et al., 2010, 2012). In our studies, the strain background is controlled and thus, also is the potential for susceptibility defined by this background. As such, it is unlikely that differences in disease outcomes noted in our *IL-6*-/- mice are attributable to the 0.7 mmHg difference in absolute IOP and likely arise from other IL-6-dependent outcomes.

Second, baseline axon density in the optic nerve of *IL-6*-/- mice was approximately 15% lower than that of WT mice. Our assessment of axon density in PPD-stained semi-thin sections allows counting of only myelinated axons. Previous literature documents that IL-6 influences oligodendrocyte differentiation and gene expression associated with myelination *in vitro* (Valerio et al., 2002; Zhang et al., 2006, 2007). As such, it is probable that myelination of RGC axons is altered in our *IL-6*-/- mice. This may or may not have consequences beyond confounding axon density measurements. Further studies with conditional perturbation of IL-6 signaling will be needed to elucidate the source and impact of confounds arising from developmental deficiency of IL-6 signaling.

In conclusion, our findings indicate that IL-6 is part of a mechanism that specifically leads to structural degeneration of axons. Furthermore, its absence is sufficient to prevent both structural degeneration of the optic nerve and vision loss. That the functional and structural components of RGC axonopathy could be mechanistically separated has tremendous implications for therapeutic targeting, and our findings identify IL-6 as a potential candidate for such.

## Author contributions

FE designed the study, performed experiments, analyzed data and wrote the manuscript. CF performed experiments and reviewed the manuscript. RS designed the study, analyzed data and revised the manuscript. All authors have read and approved the final manuscript.

### Conflict of interest statement

The authors declare that the research was conducted in the absence of any commercial or financial relationships that could be construed as a potential conflict of interest.

## Abbreviations

IL-6: interleukin-6
IOP: intraocular pressure
CNS: central nervous system
RGC: retinal ganglion cell
GCL: ganglion cell layer
NFL: nerve fiber layer
SC: superior colliculus
GFAP: glial fibrillary acidic protein
Iba-1: ionized calcium-binding adapter molecule-1
CTB: cholera toxin beta-subunit
SD-OCT: spectral domain optical coherence tomography.

## References

[B1] Birke M. T. Birke K. Lutjen-Drecoll E. Schlotzer-Schrehardt U. Hammer C. M. (2011). Cytokine-dependent ELAM-1 induction and concomitant intraocular pressure regulation in porcine anterior eye perfusion culture. Invest. Ophthalmol. Vis. Sci. 52, 468–475. 10.1167/iovs.10-5990 20861478

[B2] Blindenbacher A. Wang X. Langer I. Savino R. Terracciano L. Heim M. H. (2003). Interleukin 6 is important for survival after partial hepatectomy in mice. Hepatology 38, 674–682. 10.1053/jhep.2003.50378 12939594

[B3] Bluthe R. M. Michaud B. Poli V. Dantzer R. (2000). Role of IL-6 in cytokine-induced sickness behavior: a study with IL-6 deficient mice. Physiol. Behav. 70, 367–373. 10.1016/S0031-9384(00)00269-9 11006436

[B4] Bond W. S. Hines-Beard J. GoldenMerry Y. P. Davis M. Farooque A. Sappington R. M. (2016). Virus-mediated EpoR76E therapy slows optic nerve axonopathy in experimental glaucoma. Mol. Ther. 24, 230–239. 10.1038/mt.2015.198 PMC4817814 26502777

[B5] Bosco A. Inman D. M. Steele M. R. Wu G. Soto I. Marsh-Armstrong N.. (2008). Reduced retina microglial activation and improved optic nerve integrity with minocycline treatment in the DBA/2J mouse model of glaucoma. Invest. Ophthalmol. Vis. Sci. 49, 1437–1446. 10.1167/iovs.07-1337 18385061

[B6] Bosco A. Steele M. R. Vetter M. L. (2011). Early microglia activation in a mouse model of chronic glaucoma. J. Comp. Neurol. 519, 599–620. 10.1002/cne.22516 21246546 PMC4169989

[B7] Burton M. D. Johnson R. W. (2012). Interleukin-6 trans-signaling in the senescent mouse brain is involved in infection-related deficits in contextual fear conditioning. Brain Behav. Immun. 26, 732–738. 10.1016/j.bbi.2011.10.008 22062497 PMC3699311

[B8] Burton M. D. Rytych J. L. Freund G. G. Johnson R. W. (2013). Central inhibition of interleukin-6 trans-signaling during peripheral infection reduced neuroinflammation and sickness in aged mice. Brain Behav. Immun. 30, 66–72. 10.1016/j.bbi.2013.01.002 23354002 PMC3641158

[B9] Burton M. D. Sparkman N. L. Johnson R. W. (2011). Inhibition of interleukin-6 trans-signaling in the brain facilitates recovery from lipopolysaccharide-induced sickness behavior. J. Neuroinflammation 8:54. 10.1186/1742-2094-8-54 21595956 PMC3113341

[B10] Calkins D. J. (2012). Critical pathogenic events underlying progression of neurodegeneration in glaucoma. Prog. Retin. Eye Res. 31, 702–719. 10.1016/j.preteyeres.2012.07.001 22871543 PMC3472111

[B11] Campbell I. L. Abraham C. R. Masliah E. Kemper P. Inglis J. D. Oldstone M. B.. (1993). Neurologic disease induced in transgenic mice by cerebral overexpression of interleukin 6. Proc. Natl. Acad. Sci. U.S.A. 90, 10061–10065. 10.1073/pnas.90.21.10061 7694279 PMC47713

[B12] Cardenas H. Bolin L. M. (2003). Compromised reactive microgliosis in MPTP-lesioned IL-6 KO mice. Brain Res. 985, 89–97. 10.1016/S0006-8993(03)03172-X 12957371

[B13] Chidlow G. Wood J. P. Ebneter A. Casson R. J. (2012). Interleukin-6 is an efficacious marker of axonal transport disruption during experimental glaucoma and stimulates neuritogenesis in cultured retinal ganglion cells. Neurobiol. Dis. 48, 568–581. 10.1016/j.nbd.2012.07.026 22884876

[B14] Chucair-Elliott A. J. Conrady C. Zheng M. Kroll C. M. Lane T. E. Carr D. J. (2014). Microglia-induced IL-6 protects against neuronal loss following HSV-1 infection of neural progenitor cells. Glia 62, 1418–1434. 10.1002/glia.22689 24807365 PMC4107000

[B15] Clark W. M. Rinker L. G. Lessov N. S. Hazel K. Hill J. K. Stenzel-Poore M.. (2000). Lack of interleukin-6 expression is not protective against focal central nervous system ischemia. Stroke 31, 1715–1720. 10.1161/01.STR.31.7.1715 10884478

[B16] Cone F. E. Gelman S. E. Son J. L. Pease M. E. Quigley H. A. (2010). Differential susceptibility to experimental glaucoma among 3 mouse strains using bead and viscoelastic injection. Exp. Eye Res. 91, 415–424. 10.1016/j.exer.2010.06.018 20599961 PMC2954410

[B17] Cone F. E. Steinhart M. R. Oglesby E. N. Kalesnykas G. Pease M. E. Quigley H. A. (2012). The effects of anesthesia, mouse strain and age on intraocular pressure and an improved murine model of experimental glaucoma. Exp. Eye Res. 99, 27–35. 10.1016/j.exer.2012.04.006 22554836 PMC3375133

[B18] Crish S. D. Dapper J. D. MacNamee S. E. Balaram P. Sidorova T. N. Lambert W. S.. (2013). Failure of axonal transport induces a spatially coincident increase in astrocyte BDNF prior to synapse loss in a central target. Neuroscience 229, 55–70. 10.1016/j.neuroscience.2012.10.069 23159315 PMC3534890

[B19] Crish S. D. Sappington R. M. Inman D. M. Horner P. J. Calkins D. J. (2010). Distal axonopathy with structural persistence in glaucomatous neurodegeneration. Proc. Natl. Acad. Sci. U.S.A. 107, 5196–5201. 10.1073/pnas.0913141107 20194762 PMC2841892

[B20] De Moraes C. G. Juthani V. J. Liebmann J. M. Teng C. C. Tello C. Susanna R. Jr.. (2011). Risk factors for visual field progression in treated glaucoma. Arch. Ophthalmol. 129, 562–568. 10.1001/archophthalmol.2011.72 21555607

[B21] Douglas R. M. Alam N. M. Silver B. D. McGill T. J. Tschetter W. W. Prusky G. T. (2005). Independent visual threshold measurements in the two eyes of freely moving rats and mice using a virtual-reality optokinetic system. Vis. Neurosci. 22, 677–684. 10.1017/S0952523805225166 16332278

[B22] Du S. Huang W. Zhang X. Wang J. Wang W. Lam D. S. (2016). Multiplex cytokine levels of aqueous humor in acute primary angle-closure patients: fellow eye comparison. BMC Ophthalmol. 16:6. 10.1186/s12886-016-0182-8 26748993 PMC4707003

[B23] Echevarria F. D. Rickman A. E. Sappington R. M. (2016). Interleukin-6: a constitutive modulator of glycoprotein 130, neuroinflammatory and cell survival signaling in retina. J. Clin. Cell. Iummunol. 7:439. 10.4172/2155-9899.1000439 27747134 PMC5061045

[B24] Echevarria F. Walker C. Abella S. Won M. Sappington R. (2013). Stressor-dependent alterations in glycoprotein 130: implications for glial cell reactivity, cytokine signaling and ganglion cell health in glaucoma. J. Clin. Exp. Ophthalmol. 4:1000286. 10.4172/2155-9570.1000286 25018894 PMC4091850

[B25] Engel L. A. Muether P. S. Fauser S. Hueber A. (2014). The effect of previous surgery and topical eye drops for primary open-angle glaucoma on cytokine expression in aqueous humor. Graefes Arch. Clin. Exp. Ophthalmol. 252, 791–799. 10.1007/s00417-014-2607-5 24638257

[B26] Erta M. Quintana A. Hidalgo J. (2012). Interleukin-6, a major cytokine in the central nervous system. Int. J. Biol. Sci. 8, 1254–1266. 10.7150/ijbs.4679 23136554 PMC3491449

[B27] Fang X. X. Jiang X. L. Han X. H. Peng Y. P. Qiu Y. H. (2013). Neuroprotection of interleukin-6 against NMDA-induced neurotoxicity is mediated by JAK/STAT3, MAPK/ERK, and PI3K/AKT signaling pathways. Cell. Mol. Neurobiol. 33, 241–251. 10.1007/s10571-012-9891-6 23161148 PMC11497919

[B28] Fisher J. Mizrahi T. Schori H. Yoles E. Levkovitch-Verbin H. Haggiag S.. (2001). Increased post-traumatic survival of neurons in IL-6-knockout mice on a background of EAE susceptibility. J. Neuroimmunol. 119, 1–9. 10.1016/S0165-5728(01)00342-3 11525794

[B29] Formichella C. Abella S. K. Sims S. M. Cathcart H. M. Sappington R. M. (2014). Astrocyte reactivity: a biomarker for ganglion cell health in retinal neurodegeneration. J. Clin. Cell. Immunol. 5:15. 10.4172/2155-9899.1000188 25133067 PMC4131747

[B30] Gallego B. I. Salazar J. J. de Hoz R. Rojas B. Ramirez A. I. Salinas-Navarro M.. (2012). IOP induces upregulation of GFAP and MHC-II and microglia reactivity in mice retina contralateral to experimental glaucoma. J. Neuroinflammation 9:92. 10.1186/1742-2094-9-92 22583833 PMC3410794

[B31] Gao S. Jakobs T. C. (2016). Mice homozygous for a deletion in the glaucoma susceptibility locus INK4 show increased vulnerability of retinal ganglion cells to elevated intraocular pressure. Am. J. Pathol. 186, 985–1005. 10.1016/j.ajpath.2015.11.026 26883755 PMC5848263

[B32] Hines-Beard J. Bond W. S. Backstrom J. R. Rex T. S. (2016). Virus-mediated EpoR76E gene therapy preserves vision in a glaucoma model by modulating neuroinflammation and decreasing oxidative stress. J. Neuroinflammation 13:39. 10.1186/s12974-016-0499-5 26876380 PMC4753658

[B33] Huang W. Chen S. Gao X. Yang M. Zhang J. Li X.. (2014). Inflammation-related cytokines of aqueous humor in acute primary angle-closure eyes. Invest. Ophthalmol. Vis. Sci. 55, 1088–1094. 10.1167/iovs.13-13591 24474267

[B34] Inman D. M. Horner P. J. (2007). Reactive nonproliferative gliosis predominates in a chronic mouse model of glaucoma. Glia 55, 942–953. 10.1002/glia.20516 17457855

[B35] Inomata Y. Hirata A. Yonemura N. Koga T. Kido N. Tanihara H. (2003). Neuroprotective effects of interleukin-6 on NMDA-induced rat retinal damage. Biochem. Biophys. Res. Commun. 302, 226–232. 10.1016/S0006-291X(03)00127-X 12604335

[B36] Johnson E. C. Doser T. A. Cepurna W. O. Dyck J. A. Jia L. Guo Y.. (2011). Cell proliferation and interleukin-6-type cytokine signaling are implicated by gene expression responses in early optic nerve head injury in rat glaucoma. Invest. Ophthalmol. Vis. Sci. 52, 504–518. 10.1167/iovs.10-5317 20847120 PMC3053294

[B37] Johnson E. C. Morrison J. C. (2009). Friend or foe? Resolving the impact of glial responses in glaucoma. J. Glaucoma 18, 341–353. 10.1097/IJG.0b013e31818c6ef6 19525723 PMC2697444

[B38] Kopf M. Baumann H. Freer G. Freudenberg M. Lamers M. Kishimoto T.. (1994). Impaired immune and acute-phase responses in interleukin-6-deficient mice. Nature 368, 339–342. 10.1038/368339a0 8127368

[B39] Lambert W. S. Ruiz L. Crish S. D. Wheeler L. A. Calkins D. J. (2011). Brimonidine prevents axonal and somatic degeneration of retinal ganglion cell neurons. Mol. Neurodegener. 6:4. 10.1186/1750-1326-6-4 21232114 PMC3035592

[B40] Leibinger M. Andreadaki A. Gobrecht P. Levin E. Fischer D. (2016). Boosting CNS axon regeneration by circumventing limitations of natural cytokine signaling. Mol. Ther. 4, 1712–1725. 10.1038/mt.2016.102 PMC5112033 27203446

[B41] Leibinger M. Muller A. Gobrecht P. Diekmann H. Andreadaki A. Fischer D. (2013). Interleukin-6 contributes to CNS axon regeneration upon inflammatory stimulation. Cell Death Dis. 4:e609. 10.1038/cddis.2013.126 23618907 PMC3641349

[B42] Lin Z. Q. Kondo T. Ishida Y. Takayasu T. Mukaida N. (2003). Essential involvement of IL-6 in the skin wound-healing process as evidenced by delayed wound healing in IL-6-deficient mice. J. Leukoc. Biol. 73, 713–721. 10.1189/jlb.0802397 12773503

[B43] Loddick S. A. Turnbull A. V. Rothwell N. J. (1998). Cerebral interleukin-6 is neuroprotective during permanent focal cerebral ischemia in the rat. J. Cereb. Blood Flow Metab. 18, 176–179. 10.1097/00004647-199802000-00008 9469160

[B44] Lye-Barthel M. Sun D. Jakobs T. C. (2013). Morphology of astrocytes in a glaucomatous optic nerve. Invest. Ophthalmol. Vis. Sci. 54, 909–917. 10.1167/iovs.12-10109 23322566 PMC3564449

[B45] Martin A. Hofmann H. D. Kirsch M. (2003). Glial reactivity in ciliary neurotrophic factor-deficient mice after optic nerve lesion. J. Neurosci. 23, 5416–5424. 12843240 10.1523/JNEUROSCI.23-13-05416.2003 PMC6741243

[B46] McFarland-Mancini M. M. Funk H. M. Paluch A. M. Zhou M. Giridhar P. V. Mercer C. A.. (2010). Differences in wound healing in mice with deficiency of IL-6 versus IL-6 receptor. J. Immunol. 184, 7219–7228. 10.4049/jimmunol.0901929 20483735

[B47] Mukaino M. Nakamura M. Yamada O. Okada S. Morikawa S. Renault-Mihara F.. (2010). Anti-IL-6-receptor antibody promotes repair of spinal cord injury by inducing microglia-dominant inflammation. Exp. Neurol. 224, 403–414. 10.1016/j.expneurol.2010.04.020 20478301

[B48] Nouri-Mahdavi K. Hoffman D. Coleman A. L. Liu G. Li G. Gaasterland D.. (2004). Predictive factors for glaucomatous visual field progression in the Advanced Glaucoma Intervention Study. Ophthalmology 111, 1627–1635. 10.1016/j.ophtha.2004.02.017 15350314

[B49] Penkowa M. Giralt M. Carrasco J. Hadberg H. Hidalgo J. (2000). Impaired inflammatory response and increased oxidative stress and neurodegeneration after brain injury in interleukin-6-deficient mice. Glia 32, 271–285. 10.1002/1098-1136(200012)32:3<271::AID-GLIA70>3.0.CO;2-5 11102968

[B50] Penkowa M. Giralt M. Lago N. Camats J. Carrasco J. Hernandez J.. (2003). Astrocyte-targeted expression of IL-6 protects the CNS against a focal brain injury. Exp. Neurol. 181, 130–148. 10.1016/S0014-4886(02)00051-1 12781987

[B51] Penkowa M. Molinero A. Carrasco J. Hidalgo J. (2001). Interleukin-6 deficiency reduces the brain inflammatory response and increases oxidative stress and neurodegeneration after kainic acid-induced seizures. Neuroscience 102, 805–818. 10.1016/S0306-4522(00)00515-7 11182244

[B52] Penkowa M. Moos T. Carrasco J. Hadberg H. Molinero A. Bluethmann H.. (1999). Strongly compromised inflammatory response to brain injury in interleukin-6-deficient mice. Glia 25, 343–357. 10.1002/(SICI)1098-1136(19990215)25:4<343::AID-GLIA4>3.0.CO;2-V 10028917

[B53] Rojas B. Gallego B. I. Ramirez A. I. Salazar J. J. de Hoz R. Valiente-Soriano F. J.. (2014). Microglia in mouse retina contralateral to experimental glaucoma exhibit multiple signs of activation in all retinal layers. J. Neuroinflammation 11:133. 10.1186/1742-2094-11-133 25064005 PMC4128533

[B54] Sappington R. M. Calkins D. J. (2006). Pressure-induced regulation of IL-6 in retinal glial cells: involvement of the ubiquitin/proteasome pathway and NFkappaB. Invest. Ophthalmol. Vis. Sci. 47, 3860–3869. 10.1167/iovs.05-1408 16936098

[B55] Sappington R. M. Calkins D. J. (2008). Contribution of TRPV1 to microglia-derived IL-6 and NFkappaB translocation with elevated hydrostatic pressure. Invest. Ophthalmol. Vis. Sci. 49, 3004–3017. 10.1167/iovs.07-1355 18362111 PMC4139938

[B56] Sappington R. M. Carlson B. J. Crish S. D. Calkins D. J. (2010). The microbead occlusion model: a paradigm for induced ocular hypertension in rats and mice. Invest. Ophthalmol. Vis. Sci. 51, 207–216. 10.1167/iovs.09-3947 19850836 PMC2869054

[B57] Sappington R. M. Chan M. Calkins D. J. (2006). Interleukin-6 protects retinal ganglion cells from pressure-induced death. Invest. Ophthalmol. Vis. Sci. 47, 2932–2942. 10.1167/iovs.05-1407 16799036

[B58] Sihota R. Saxena R. Gogoi M. Sood A. Gulati V. Pandey R. M. (2005). A comparison of the circadian rhythm of intraocular pressure in primary phronic angle closure glaucoma, primary open angle glaucoma and normal eyes. Indian J. Ophthalmol. 53, 243–247. 10.4103/0301-4738.18905 16333172

[B59] Sims S. M. Holmgren L. Cathcart H. M. Sappington R. M. (2012). Spatial regulation of interleukin-6 signaling in response to neurodegenerative stressors in the retina. Am. J. Neurodegener. Dis. 1, 168–179. 23024928 PMC3560463

[B60] Sparkman N. L. Buchanan J. B. Heyen J. R. Chen J. Beverly J. L. Johnson R. W. (2006). Interleukin-6 facilitates lipopolysaccharide-induced disruption in working memory and expression of other proinflammatory cytokines in hippocampal neuronal cell layers. J. Neurosci. 26, 10709–10716. 10.1523/JNEUROSCI.3376-06.2006 17050710 PMC6674759

[B61] Spittau B. Zhou X. Ming M. Krieglstein K. (2012). IL6 protects MN9D cells and midbrain dopaminergic neurons from MPP+-induced neurodegeneration. Neuromolecular Med. 14, 317–327. 10.1007/s12017-012-8189-7 22772723

[B62] Takai Y. Tanito M. Ohira A. (2012). Multiplex cytokine analysis of aqueous humor in eyes with primary open-angle glaucoma, exfoliation glaucoma, and cataract. Invest. Ophthalmol. Vis. Sci. 53, 241–247. 10.1167/iovs.11-8434 22159018

[B63] Tan S. Baig N. Hansapinyo L. Jhanji V. Wei S. Tham C. C. (2017). Comparison of self-measured diurnal intraocular pressure profiles using rebound tonometry between primary angle closure glaucoma and primary open angle glaucoma patients. PLoS ONE 12:e0173905. 10.1371/journal.pone.0173905 28333942 PMC5363915

[B64] Tiberio G. A. Tiberio L. Benetti A. Cervi E. Montani N. Dreano M.. (2008). IL-6 Promotes compensatory liver regeneration in cirrhotic rat after partial hepatectomy. Cytokine 42, 372–378. 10.1016/j.cyto.2008.03.012 18455423

[B65] Tojo N. Abe S. Ishida M. Yagou T. Hayashi A. (2017). The fluctuation of intraocular pressure measured by a contact lens sensor in normal-tension glaucoma patients and nonglaucoma subjects. J. Glaucoma 26, 195–200. 10.1097/IJG.0000000000000517 27552498

[B66] Valerio A. Ferrario M. Dreano M. Garotta G. Spano P. Pizzi M. (2002). Soluble interleukin-6 (IL-6) receptor/IL-6 fusion protein enhances *in vitro* differentiation of purified rat oligodendroglial lineage cells. Mol. Cell. Neurosci. 21, 602–615. 10.1006/mcne.2002.1208 12504593

[B67] Wang X. Tay S. S. W. Ng Y. K. (2000). An immunohistochemical study of neuronal and glial cell reactions in retinae of rats with experimental glaucoma. Exp. Brain Res. 132, 476–484. 10.1007/s002210000360 10912828

[B68] Ward N. J. Ho K. W. Lambert W. S. Weitlauf C. Calkins D. J. (2014). Absence of transient receptor potential vanilloid-1 accelerates stress-induced axonopathy in the optic projection. J. Neurosci. 34, 3161–3170. 10.1523/JNEUROSCI.4089-13.2014 24573275 PMC3935081

[B69] Wilson G. N. Inman D. M. Dengler Crish C. M. Smith M. A. Crish S. D. (2015). Early pro-inflammatory cytokine elevations in the DBA/2J mouse model of glaucoma. J. Neuroinflammation 12:176. 10.1186/s12974-015-0399-0 26376776 PMC4574349

[B70] Yamada M. Hatanaka H. (1994). Interleukin-6 protects cultured rat hippocampal neurons against glutamate-induced cell death. Brain Res. 643, 173–180. 10.1016/0006-8993(94)90023-X 7913397

[B71] Zhang P. L. Izrael M. Ainbinder E. Ben-Simchon L. Chebath J. Revel M. (2006). Increased myelinating capacity of embryonic stem cell derived oligodendrocyte precursors after treatment by interleukin-6/soluble interleukin-6 receptor fusion protein. Mol. Cell. Neurosci. 31, 387–398. 10.1016/j.mcn.2005.10.014 16325417

[B72] Zhang P. L. Levy A. M. Ben-Simchon L. Haggiag S. Chebath J. Revel M. (2007). Induction of neuronal and myelin-related gene expression by IL-6-receptor/IL-6: a study on embryonic dorsal root ganglia cells and isolated Schwann cells. Exp. Neurol. 208, 285–296. 10.1016/j.expneurol.2007.08.022 17963753

[B73] Zhong J. Dietzel I. D. Wahle P. Kopf M. Heumann R. (1999). Sensory impairments and delayed regeneration of sensory axons in interleukin-6-deficient mice. J. Neurosci. 19, 4305–4313. 10341234 10.1523/JNEUROSCI.19-11-04305.1999 PMC6782624

